# Evaluation on Net Energy of Defatted Rice Bran from Different Origins and Processing Technologies Fed to Growing Pigs

**DOI:** 10.3390/ani11041106

**Published:** 2021-04-12

**Authors:** Bingbing Huang, Li Wang, Zhiqian Lyu, Lu Wang, Jianjun Zang, Defa Li, Changhua Lai

**Affiliations:** State Key Laboratory of Animal Nutrition, College of Animal Science and Technology, China Agricultural University, Beijing 100193, China; bbhuang217@163.com (B.H.); q15928806587@163.com (L.W.); lzq2434390936@163.com (Z.L.); wanglucau@163.com (L.W.); zangjj@cau.edu.cn (J.Z.); defali@cau.edu.cn (D.L.)

**Keywords:** defatted rice bran, growing pigs, indirect calorimetry, net energy, process

## Abstract

**Simple Summary:**

In recent years, prices of imported staples such as corn and soybean meal have risen dramatically. Defatted rice bran (DFRB), an abundant and underutilized agricultural coproduct of the paddy rice, was a replacement of corn and soybean meal. It is necessary to comprehensively evaluate the nutritional value of DFRB. This study determined and compared the net energy (NE) of DFRB from different sources and different processing technology fed to growing pigs using indirect calorimetry. Results indicated that NE contents of extruded DFRB from different provinces were within the range of values ((8.24 to 10.22 MJ/kg dry matter (DM)). The NE contents of extruded DFRB and pelleted DFRB from the same province were 8.24 vs. 6.56 MJ/kg DM. This study showed that there is a discrepancy of approximately 10.01% in the NE content between the DFRB origins. The data above suggested that NE content of DFRB could be related to DFRB origins and processing technology. More NE contents of different DFRB samples deserve to be explored further. The study supported some theoretical foundation for the application of DFRB in the NE system.

**Abstract:**

The study was conducted to determine and compare the net energy (NE) of defatted rice bran (DFRB) from different sources and different processing technology fed to growing pigs using indirect calorimetry. Thirty-six growing barrows (30.7 ± 3.9 kg) were randomly allotted to 1 of 6 diets with 6 replicate pigs per diet. Diets included a corn-soybean meal basal diet and 5 test diets containing 30% DFRB, respectively. These five samples come from 4 different provinces (i.e., Heilongjiang, Jiangsu, Jilin, and Liaoning province within China) and two of them with the same origin but different processing technologies (i.e., extruded or pelleted). During each period, pigs were kept individually in metabolism crates for 21 days, including 14 days to adapt to the diets. On day 15, pigs were transferred to the open-circuit respiration chambers for adaptation, and the next day were ready to determine daily total heat production (HP) and were fed 1 of the 6 diets at 2.3 MJ metabolizable energy (ME)/kg body weight (BW)^0.6^/day. Total feces and urine were collected for the determination of digestible energy (DE) and ME and daily total HP was measured from day 16 to day 20 and fasted on day 21 for the measurement of fasting heat production (FHP). The NE contents of extruded DFRB from different provinces were within the range of values (8.24 to 10.22 MJ/kg DM). There is a discrepancy of approximately 10.01% in the NE content between the DFRB origins. The NE contents of extruded DFRB and pelleted DFRB from the same province were 8.24 vs. 6.56 MJ/kg DM. Retained energy (RE) and FHP of diets containing extruded DFRB and pelleted DFRB were 1105 vs. 892 kJ/kg BW^0.6^/day and 746 vs. 726 kJ/kg BW^0.6^/day respectively, and those in extruded DFRB from different origins were within the range of values (947 to 1105 kJ/kg BW^0.6^/day and 726 to 755 kJ/kg BW^0.6^/day, respectively). In conclusion, NE values are affected by origin and processing technology of DFRB.

## 1. Introduction

Under commercial feed production conditions, costs of energy ingredients account for 50% of total feed costs [1]. Therefore, it is necessary to accurately assess the real usable energy value of feed ingredients and reduce feed cost. Net energy (NE) is the energy used in the feed for the animal to maintain life and produce products, that is, the metabolizable energy of the feed minus the heat increment in the body of the feed [2]. Compared with digestible energy (DE) and metabolizable energy (ME) systems, theoretically, the NE system can provide a more accurate estimate of the dietary energy available to the animal [3,4]. Approximately 782 million tons of paddy rice are produced annually in the world [5], with most entering the human food market. The defatted rice bran (DFRB) is a by-product from the processing industry of rice or oil containing crude protein (CP), fiber, and starch, as well as contains low ether extract (EE). It can be used as a supplement for protein feed and fiber feed. Several recent studies in pigs have demonstrated that DFRB shows excellent potential for inclusion in diets as a replacement for more traditional cereals such as maize and soybean meal. For example, Herfel et al. [6] and Casas et al. [7] reported that there were no effects on growth performance when no more than 20% of DFRB was included in diets for pigs. However, the DE or ME overestimate the energy values of fiber-rich feeds and protein feeds, whereas the NE provides energy values of feeds that most closely describe the available energy of pigs because it takes the heat increment from digestive utilization and metabolism of feeds into account [3,8,9], and the NE system takes into account the heat increment. Therefore, it provides a theoretical basis for more rational use of DFRB. In our previous studies, the DE, ME, and standardized ileal digestible (SID) amino acid (AA) of different provenances DFRB were evaluated, and research has shown that the source of defatted rice bran affects the energy content [10] and few studies exist evaluating DFRB [10,11,12]. The NE of one defatted rice bran sample has been determined [13]. For all we know, there is only one published dataset for NE of DFRB in pigs. In particular, there is no study determining NE of DFRB distinguishing among processing technologies used (i.e., extruded or pelleted) and DFRB origins. 

Therefore, the objective of this experiment was to determine and compare the NE of DFRB from different origins and processing technologies fed to growing pigs.

## 2. Materials and Methods

All protocols used in this experiment were reviewed and approved by the Institutional Animal Care and Use Committee of China Agricultural University (Beijing, China; No. AW61301202-1-1).

### 2.1. Origin and Processing Technology of Defatted Rice Bran

In the current study, the DFRB samples were collected from 4 provinces within China, including Heilongjiang, Jiangsu, Jilin, and Liaoning province. In particular, Heilongjiang, Jilin, and Liaoning province are located in northeast China, and Jiangsu province is located in east China. Northeast China has a temperate monsoon climate: summer has high temperatures and rain, while winters are cold and dry, and has four distinctive seasons. Heilongjiang province: 43°26′ to 53°33′ north latitude and 121°11′ to 135°05′ east longitude. Jilin province: 40°50′ to 46°19′ north latitude and 121°38′ to 131°19′ east longitude. Liaoning province: 38°43′ to 43°26′ north latitude and 118°53′ to 125°46′ east longitude. East China has a subtropics monsoon climate, summers have high temperatures and rain, and winters are mild with less rain. The eastern coast of China: 30°45′ to 35°08′ north latitude and 116°21′ to 121°56′ east longitude. Japonica rice and indica type rice are the rice cultivars of northeast China and east China, respectively.

Two processing technologies are used for DFRB samples in this study. The extrusion method is used to expand the rice bran through an extruder. Under the action of high temperature, high pressure, and friction, the lipase in the rice bran is inactivated. In addition, after the rice bran is expanded, the moisture in the rice bran is reduced, which suppresses the rapid rise of the acid value and makes the acid value change more slowly, thereby improving the stability of the rice bran and extending the storage time of the DFRB. The temperature is up to 130 °C during extruding. The pellet method is used to moisturize the rice bran to 12~13% of water content, then press into granules, and the rice bran granules are dried, and it is ensured that the immersion moisture is 7% to 9% and the temperature is 50 to 55 °C. Both methods use solvent extraction and desolventing. 

### 2.2. Animals, Diets, and Experimental Design

Thirty-six growing barrows (Duroc × Landrace × Yorkshire) with an average initial body weight (BW) of 33.27 ± 6.67 kg were randomized and allotted to 6 diets with 6 replicates per diet. The 6 experimental diets with a constant corn:soybean meal ratio included a corn-soybean meal-based basal diet and 5 experimental diets containing 30% DFRB, which replaced 30.82% of the energy supplied by corn, soybean meal, and AA, respectively. Crystalline lysine, methionine, and threonine were added to the diets to meet the pigs’ requirements. The composition and nutrient levels of ingredients and diets are shown in Table 1 and Table 2.

The experiment was conducted in 6 consecutive periods (6 pigs per period), and each period lasted 21 days. Each period included a 14-day diet adaptation period [13] and a 6-day heat production (HP) measurement period. The pigs were individually housed in adjustable stainless-steel metabolism crates with a feeder and a nipple drinker and located in a temperature-controlled room (22 ± 2 °C) for the initial 14 days. On day 15, pigs were transferred to the metabolic cages within the open-circuit respiration chambers for environment adaptation. The next 5 days were for the collection of feces and urine, and meanwhile, daily O_2_ consumption and CO_2_ and CH_4_ productions were continuously measured. On the last day of each period (day 20), pigs were fasted: the HP measured during the last 8 h from 22:30 (day 20) to 06:30 (day 21) was considered as fasting heat production (FHP). The FHP period started 31 h after the last meal and with animals kept in the dark to minimize physical activity [14]. The period only collected urine. Pigs were fed their allocated diets at 2.3 MJ ME/kg BW^0.6^/day [15] based on BW measured on days 0, 7, and 14. Pigs were fed equal sized meals twice daily at 08:30 and 15:30 using automatic feeders and had free access to water throughout the trial. 

### 2.3. Sample Collection

Pigs were weighed on days 15, 20, and 21. The chambers were opened for approximately 1 h at 08:30 and 15:30 every day to feed pigs and collect feces. The data of O_2_ consumption and CO_2_ and CH_4_ production during this period were not included in the calculation of daily HP due to the CO_2_ concentration in the chamber decreasing when the door was opened. Therefore, the calculation of HP began when the CO_2_ concentration in the chamber was above 2000 ppm [16]. Six chambers were used in the present experiment, and more details on the construction of chambers and indirect calorimetry method were described by van Milgen et al. [17] and Zhang et al. [15]. The temperature was maintained at 22 °C during the fed state and 24 °C during the fasted state. The relative humidity was controlled close to 70%. A 10 h (from 08:00 to 18:00) lighting schedule was used per day. The gas analyzers were reported by Lyu et al. [13].

During each period, feed refusals and spillage were collected once daily and subsequently dried, weighed, and recorded. From day 15 to day 20, feces and urine were collected once daily at 08:30 according to the total collection procedure. All feces were collected immediately and stored at −20 °C. Only urine was collected in the fasted period for determination of FHP. The urine of the fed period and fasted period were stored separately. Urine was collected using plastic containers which contained 50 mL of 6 ***N*** HCl to limit microbial growth and reduce ammonia loss [18]. The weights of collected feces were recorded every day. Urine volume measured as weight was determined each day, and a 10% daily aliquot was then quickly stored at −20 °C.

All DFRB samples were collected at the preparation of diets in order to measure the dry matter (DM). Diets were collected after preparation to analyze chemical composition. At the end of the experiment, fecal samples from each pig in each period were thawed, mixed, weighed, and a 350 g sample was taken and dried in a forced draft oven at 65 °C for 72 h. After drying and grinding through a 1 mm screen, subsamples were stored at −20 °C until being used for further chemical analysis. Urine samples were thawed and thoroughly mixed, and a sub-sample was collected for analysis. Urine samples (4 mL) were dried at 65 °C for 8 h with quantitative filter papers in crucibles for energy determination [18]. Urine samples of the fasted period only determined N loss.

### 2.4. Chemical Analyses

The DM analyses of DFRB, diets, and feces were determined by using the forced-air oven (model GZX-9140 MBE; Boxun Company, Shanghai, China) at 105 °C for 6 h to a constant weight (method 934.01) [19]. The Kjeldahl method (Foss Kjeltec^TM 2100^; Foss Kemao Inc., Beijing, China) was used for nitrogen in DFRB, diets, feces, and urine samples (method 984.13) [19]. The CP was calculated as nitrogen × 6.25. The gross energy (GE) in the DFRB, diets, feces, and urine samples were determined using an Automatic Isoperibol Oxygen Bomb Calorimeter (Parr 6300 Calorimeter; Parr Instrument Company, Moline, IL, USA). The neutral detergent fiber (NDF) and acid detergent fiber (ADF) in DFRB, diets, and feces samples were determined using a fiber analyzer (ANKOM^200^ fiber analyzer; ANKOM Technology, Macedon, NY, USA). The ash in DFRB, diets, and feces samples were analyzed by burning carbonized samples at 550 °C for 8 h until light gray ash results, or to constant weight (method 923.03) [19]. The insoluble dietary fiber (IDF) and soluble dietary fiber (SDF) contents of DFRB were determined using IDF bags and SDF bags (Dietary Fiber Analysis-IDF/SDF; ANKOM Technology, Macedon, NY, USA). The total dietary fiber (TDF) was calculated by adding the values of IDF and SDF. The phosphorus in the DFRB was analyzed according to the vanadate colorimetric method (method 946.06) [19] using a spectrophotometer (Spectrumlab 721 s; Lengguang Company, Shanghai, China). The DFRB samples were analyzed for calcium (method 968.08) [19] by an atomic absorption spectrometer (Hitachi Z-2000; Hitachi Ltd., Tokyo, Japan). Total starch content of DFRB was measured according to American Association of Cereal Chemists [20].

The AA (method 982.30) contents in ingredients were analyzed according to the procedures of Association of Official Analytical Chemists [19]. Samples were hydrolyzed with 6 ***N*** HCl at 110 °C for 24 h and then analyzed for 15 AA using an Amino Acid Analyzer (Hitachi L-8900, Tokyo, Japan). Methionine and cystine were determined as methionine sulfone and cysteic acid after cold performic acid oxidation overnight and hydrolyzing with 7.5 ***N*** HCl at 110 °C for 24 h [21,22] before measurement using an Amino Acid Analyzer (Hitachi L-8800, Tokyo, Japan). Tryptophan was determined after hydrolysis with LiOH for 22 h at a constant temperature of 110 °C [23] and then analyzed using high-performance liquid chromatography (Agilent 1200 Series, Santa Clara, CA, USA).

### 2.5. Calculations

During each collection period (from day 16 to day 20), the DM intake in the feed was calculated as the product of feed intake and DM content of diets. Gross energy intake was calculated as the product of the actual feed DM intake and the GE content of diets. The energy lost in methane, feces, and urine were measured for each pig per day. The ME content was calculated by subtracting energy in the methane and urine from DE. Energy lost as methane was calculated using the 39.54 kJ/L conversion factor [24].

The apparent total tract digestibility (ATTD) of energy and nutrients were calculated according to Adeola [25] using the following equation: ATTD (%) = ((total intake of energy (kJ) or nutrients (g) − total fecal output of energy (kJ) or nutrients (g))/total intake of energy (kJ) or nutrients (g)) × 100%.

During each period, the concentration of O_2_, CO_2_, and CH_4_ in both ingoing and outgoing air, and outgoing air flow rates were measured at 5 min intervals. Then, these values were averaged and extrapolated to a 24 h period. Total HP was then calculated for each day by gas exchange volumes and urinary loss of N according to Brouwer [24] using the following equation: HP (kJ) = 16.18 × O_2_ (L) + 5.02 × CO_2_ (L) − 2.17 × CH_4_ (L) − 5.99 × urinary N (g).

The FHP was calculated using the same equation for total HP with gas concentrations and air flow obtained from the last 8 h HP measurement from day 20 to day 21 (i.e., from 22:00 to 06:00). For base production using the same time span as used for total HP, the 8 h HP was then extrapolated to a 24 h period.

Retained energy (RE) was calculated according to the following equation [15]: RE (kJ/kg DM) = (ME intake (kJ/day) − HP (kJ/day))/DM intake (kg/day).

Retention of energy as protein (RE_P_) was calculated as N retention (g) × 6.25 × 23.86 (kJ/g). Retention of energy as lipid (RE_L_) was calculated as the difference between RE and RE_P_. 

Net energy of each diet was calculated according to Noblet et al. [15] using the following equation: NE (kJ/kg DM) = (RE (kJ/day) + FHP (kJ/day))/DM intake (kg/day). 

The DM of minerals and vitamins in the basal diet was 2.97%, which was considered to not provide any energy; therefore, the DE, ME, and NE of the basal diet were divided by 97.03% (the DM ratio of corn, soybean meal, and AA in the basal diet) to calculate the DE, ME, and NE of the corn and soybean meal mixture. The average GE, DE, ME, and NE contributions of each ingredient from the mean GE, DE, ME, and NE contents of each diet were calculated by the difference method [25]. We assumed that the average GE, DE, ME, and NE of the corn and soybean meal and AA mixture obtained for the basal diet was applied to the other experimental diets. The DE:GE, ME:DE, and NE:ME ratios could then be calculated for ingredients and used to estimate the final DE, ME, and NE values. The DE in each ingredient was calculated as GE measured and DE:GE ratio, the ME in each ingredient was calculated as measured GE and DE:GE and ME:DE ratios, and NE in each ingredient was calculated as measured GE and DE:GE, ME:DE, and NE:ME ratio. All calculations were based on DM. The respiratory quotient (RQ) was calculated as the ratio between CO_2_ production and O_2_ consumption.

### 2.6. Statistical Analyses

All data for this experiment were analyzed using the General liner model procedure of SAS 9.2 (SAS Institute Inc., Carry, NC, USA) for a completely randomized design with individual pig as the experimental unit. The UNIVARIATE procedure of SAS 9.2 (SAS Inst. Inc., Cary, NC, USA) was used to check the normality of residuals and equal variances. Diet was treated as the only fixed effect, and period and chamber as random effects. Statistical differences among the treatments were separated by Duncan’s multiple range test. Treatment means were calculated using the LSMEANS statement and statistical significance was declared at *p* < 0.05.

## 3. Results

All pigs adapted well to environmental conditions and their diets, readily consumed their daily feed allowance, and remained healthy during the experiment. At the beginning of the animal trial, the average BW of the pigs was 33.27 ± 6.67 kg, and the average BW of the pigs at the end was 37.59 ± 7.21 kg and 35.07 ± 7.74 kg before and after the FHP measurement period.

### 3.1. Chemical Composition of Ingredients and Experimental Diets

The analyzed nutrient composition of ingredients is shown in Table 1, and ingredients and chemical composition of the experimental diets is shown in Table 2. All these analyzed values are similar to the expected values for the experimental design. These five samples come from four different provinces and two of them with the same origin but different processing technologies. The concentration of TDF, SDF, and IDF in the four extruded DFRB varied from 27.95% to 31.8%, 2.75% to 3.7%, and 24.65% to 28.1%. The TDF, SDF, and IDF content in extruded DFRB (Heilongjiang, China) was greater than those in pelleted DFRB (27.95% vs. 35.1%, 3.3% vs. 1.85%, and 24.65% vs. 33.25%). The ratio of SDF to IDF was lower in pelleted DFRB compared with extruded DFRB. Compared with Heilongjiang, Jiangsu, and Liaoning province, the DFRB from Jilin province with the same technology had a greater EE content. The analyzed content of EE in extruded DFRB was greater than that in pelleted DFRB from the same province (Heilongjiang, China). The NDF and ADF in pelleted DFRB were greater than that in extruded DFRB (33.38% vs. 21.82%, 15.55% vs. 9.39%). The starch in extruded DFRB from different sources was similar. The starch content in pelleted DFRB was lowest (18.53%). The concentration of ash in extruded DFRB ranged from 9.69% to 10.61%. Compared with extruded DFRB, pelleted DFRB contained the greatest ash content. The content of CP and GE in pelleted DFRB was lower than those in extruded DFRB. DFRB from Jiangsu province had a greater GE, CP, TDF, SDF, IDF, NDF, and ADF content but contained the lowest ash compared to that in DFRB from other provinces. Among the 5 test ingredients, the values of AA concentration were similar. The pelleted DFRB diets contained greater NDF, ADF, and ash contents than other test diets, which are in accordance with the results of NDF, ADF, and ash contents in ingredients. In addition, the NDF, ADF, and ash content was the lowest in the basal diet.

### 3.2. Energy and Nitrogen Utilization of Diets

The effects of diets on digestibility coefficients and energy and nitrogen balances of growing pigs are shown in Table 3 and Table 4. The ATTD of DM, GE, CP, and organic matter (OM) were the greatest (*p* < 0.05) in pigs fed the basal diet. The ATTD of DM (75.39%), GE (78.19%), and OM (78.85%) in the pelleted DFRB (Heilongjiang, China) diet and ATTD of CP (76.56%) in the extruded DFRB (Jiangsu, China) was lower (*p* < 0.05) when compared with the basal diets. However, there were no significant differences in ATTD of NDF and ADF among the basal and 5 DFRB diets. All ATTD of nutrients in diet were not affected by processing technology in this experiment. 

Fecal nitrogen output in the basal diet was lower (*p* < 0.05) than extruded DFRB diets (from Heilongjiang and Jiangsu province), and fiber content was higher in DFRB diets. Retained nitrogen content was not affected by dietary treatments and averaged 25.46 g/day.

The total HP (average 1212 kJ/kg BW^0.6^/day) and FHP (average 747 kJ/kg BW^0.6^/day) were not affected by dietary treatments. There were no significant differences for total RE, RE_P_, and RE_L_ when pigs were fed experimental diets. The RQ was not affected by dietary treatments but was markedly lower in the FHP period compared to fed state (0.81 vs. 1.08). The ME:DE ratio (93.67%) in extruded DFRB from Jilin province was lower (*p* = 0.05) than that in other diets. There was no significant difference in NE:ME ratio (average 80.36%) among experimental diets. The lower (*p* < 0.01) DE and ME values were observed in the high fiber diets (especially in pelleted DFRB). No differences were observed for the NE among experimental diets.

### 3.3. Apparent Total Tract Digestibility of Nutrients and Energy Content for Ingredients

Apparent total tract digestibility of nutrients and energy content of the five ingredients are shown in Table 5. The ATTD of OM of the 5 ingredients was quite variable with the lowest value for pelleted DFRB (36.86%), and other DFRB values ranged between 48% and 58%. The ATTD of ADF in pelleted DFRB was lowest, which contained the highest dietary fiber contents. In addition, as a consequence of high dietary fiber contents and its low digestibility, the ATTD of GE, CP, and OM was also lowest in the pelleted DFRB sample. In terms of origin, DFRB from Jiangsu province contained the lowest ATTD of CP (58.35%) and NDF (69.75%) compared with extruded DFRB from other provinces.

The ME:DE ratio and NE:ME ratio among the 5 ingredients averaged 94.4% and 81.0%, respectively. The DE value of the four extruded DFRB from different provinces ranged from 11.17 to 13.90 MJ/kg DM, the ME concentration ranged from 10.76 to 12.95 MJ/kg DM, and the NE concentration ranged from 8.24 to 10.17 MJ/kg DM. The value of DE, ME, and NE in extruded DFRB (Heilongjiang, China) was greater when compared with pigs fed pelleted DFRB (Heilongjiang, China; 11.17 vs. 8.16 MJ/kg DM, 10.76 vs. 8.02 MJ/kg DM, 8.24 vs. 6.56 MJ/kg DM, respectively).

## 4. Discussion

The variation in the nutrient composition may be due to the variation in sources and processing conditions. The content of nutrients in extruded DFRB from different sources was mainly affected by the rice cultivar [26,27,28]. The content of GE, CP, EE, starch, and SDF in pelleted DFRB was lower than those in extruded DFRB, whereas TDF, IDF, NDF, ADF, and ash content were greater. The result of the variation may be caused by different processing temperatures in the different factories, which may lead to Maillard reactions of different magnitudes [29]. The nutrient composition of DFRB is almost in agreement with published values [10,11,12,30]. Especially, the content of EE in the current study was lower than that reported by NRC (3.52%) [30], but NRC did not distinguish among processing technologies used (i.e., extruded or pelleted). The content of ash in the current study was greater than that reported by Wang et al. (8.9%) [12]. The starch determined in the current study was less than the value in the study of Wang et al. (37.7%) [12] and the starch concentration in pelleted DFRB is similar to the value reported by Casas and Stein [11]. The variation above may be caused by the varied processing technology and rice ecotypes. The analyzed concentration of CP and AA in DFRB agree with previous reports [10,11,30,31].

The dietary fiber content had negative effects on ATTD of DM, GE, CP, and OM [1,32]. The ATTD of GE, CP, ADF, and OM was the lowest in pelleted DFRB among five DFRB samples. The energy digestibility of a high-fiber diet was the lowest in connection with low dietary fiber digestibility in pigs [1,33]. Dietary fiber may reduce the digestibility of energy-yielding substrates such as starch, lipid, and protein, resulting in a decrease in energy digestibility [34]. On the other hand, pelleted DFRB contains relatively high concentrations of insoluble dietary fiber, which reduces digesta passage rate, thereby allowing less exposure time for digestive enzymes and nutrients [34]. The ATTD of CP in extruded DFRB diet (Jiangsu, China) was the lowest, which is supposed to be the opposite of the greatest CP concentration in the DFRB sample. It can be explained by the theory that starches in grains and legumes are always present in combination with proteins, and many of which are relatively hydrophobic. In addition, the protein-starch network is surrounded by cell walls, which may severely limit the digestibility of CP and other nutrients in some types of plant materials, and the phenomenon may result in more fecal nitrogen output. The average nitrogen retention (25.46 g/day) was similar to the value reported by Quiniou et al. [35], who indicated that the maximum amount of nitrogen retention was 24.2 g/day for growing pigs. The nitrogen retention was not affected by dietary treatment, which is consistent with the findings of Le Bellego et al. [36] and Lyu et al. [13]. It also confirmed that the AA composition of DFRB was relatively balanced. 

The HP was not affected by dietary treatment and was consistent with previous studies [37,38]. However, different data were obtained and rendered by Rijnen et al. [39] and Jaworski et al. [40]. Noblet and van Milgen [3] indicated that diets that are rich in fiber may affect physical activity and thereby affect HP. The value for FHP (747 kJ/kg BW^0.6^/day) is in agreement with the calculated value (750 kJ/kg BW^0.6^/day) of Noblet et al. [15]. Different methodologies were used to measure FHP. Noblet et al. [15] measured HP on each pig at 2 feeding levels consecutively. FHP (750 kJ/kg BW^0.6^/day) was then calculated by regression of HP on ME intake and extrapolation to zero feed intake. The FHP in the current study was lower than the value (787 kJ/kg BW^0.6^/day) of a previous study using the same method [13], but both were not affected by dietary treatment, which is consistent with the results reported by previous studies [4,41].

The DE and ME of extruded DFRB diet (Jiangsu, China) were lower (*p* < 0.01) than that of basal diets. The ME of pelleted DFRB diet (Heilongjiang, China) was also lower (*p* < 0.01) than that of basal diets. The results may be partly related to the concentration of fibrous fractions in the DFRB samples [10]. The NE:ME ratio of the DFRB diets was higher than the values (80.36%) reported by Lyu et al. [13]. The NE was also greater than the data from Lyu et al. [13]. The NE of diets was calculated as the sum of retained energy and FHP, and the retained energy determined by the current experiment was greater than the value (875 kJ/kg BW^0.6^/day) determined by Lyu et al. [13]. It is known that RE is defined as the energy stored in body tissues or secretions, accounting for 50% to 60% of the NE content of diets for growing pigs [15,38,41]. Therefore, the NE content and NE:ME ratio are based mostly on the estimate of RE.

The DE and ME in DFRB were within the range of values (8.53 to 13.21 MJ/kg DM and 8.08 to 12.32 MJ/kg DM, respectively) reported by our previous study [10]. However, the DE and ME for DFRB in this current study were higher than the values (7.79 and 7.58 MJ/kg DM, respectively) reported by Lyu et al. [13]. The difference suggested that DFRB nutritional values were varied. The DE, ME, and NE values of the four extruded DFRB from different provinces were varied. The result showed that there is a discrepancy of approximately 10.01% in the NE content between the DFRB origins. It may be due to different origins’ environments affecting paddy rice growth and nutrient concentrations. The DE, ME, and NE in pigs fed pelleted DFRB (8.16, 8.02, and 6.56 MJ/kg DM, respectively) was lower when compared with pigs fed extruded DFRB (11.17, 10.76m and 8.24 MJ/kg DM, respectively) from the same province. The pelleted DFRB had the lowest energy content result from the lowest EE and starch content but the highest fiber content. The differences confirmed that lipids and starch had greater energetic efficiency [42] than dietary fiber. The extruded DFRB from Jilin province had the greatest energy content, which was also attributed to the above-mentioned reasons. The NE of DFRB was remarkably greater than the value (4.60 MJ/kg DM) reported by Lyu et al. [13]. It is likely that the NE values of diets and ingredients could differ due to genetics, methodologies used to measure RE, feeding strategies, and environmental conditions in which pigs are kept [43].

## 5. Conclusions

The NE contents of extruded DFRB from different provinces were within the range of values 8.24 to 10.22 MJ/kg DM. The NE contents of extruded DFRB and pelleted DFRB from the same province were 8.24 vs. 6.56 MJ/kg DM. This study showed that there is a discrepancy of approximately 10.01% in the NE content between the DFRB origins. The data above suggested that the NE content of DFRB could be related to DFRB origin and processing technology.

## Figures and Tables

**Table 1:** Analyzed chemical composition of defatted rice bran (DFRB) (as-fed basis).

Items, %	Origin ^1^	Heilongjiang	Heilongjiang	Jiangsu	Jilin	Liaoning
	Process	Extruded	Pelleted	Extruded	Extruded	Extruded
Gross energy, MJ/kg		15.86	15.42	16.37	16.36	15.93
Dry matter		90.39	90.66	91.34	91.12	90.46
Crude protein		15.79	15.03	16.70	16.16	16.16
Ether extract		0.69	0.58	0.70	1.67	0.71
Starch		29.71	18.53	29.77	29.38	29.11
Total dietary fiber		27.95	35.10	31.80	28.55	29.63
Soluble dietary fiber		3.30	1.85	3.70	2.75	2.98
Insoluble dietary fiber		24.65	33.25	28.10	25.80	26.65
SDF/IDF ^2^		13.39	5.56	13.17	10.66	11.18
Neutral detergent fiber		21.82	33.38	25.05	23.76	24.22
Acid detergent fiber		9.39	15.55	10.44	8.93	9.82
Ash		10.61	13.08	9.69	10.21	10.14
Calcium		0.12	0.14	0.13	0.11	0.18
Total phosphorus		2.06	1.79	1.92	1.94	2.06
Indispensable amino acid						
Arginine		1.16	1.10	1.14	1.14	1.14
Histidine		0.38	0.41	0.42	0.40	0.41
Leucine		1.11	1.13	1.21	1.15	1.15
Isoleucine		0.55	0.56	0.60	0.58	0.57
Lysine		0.82	0.85	0.85	0.83	0.89
Methionine		0.32	0.32	0.33	0.34	0.35
Phenylalanine		0.67	0.68	0.74	0.71	0.70
Threonine		0.63	0.65	0.66	0.64	0.64
Tryptophane		0.18	0.17	0.16	0.17	0.17
Valine		0.86	0.90	0.92	0.88	0.89
Dispensable amino acid						
Alanine		0.97	1.01	1.01	1.00	1.00
Aspartic acid		1.49	1.51	1.61	1.53	1.56
Cysteine		0.33	0.31	0.34	0.33	0.33
Glutamic acid		2.13	2.10	2.25	2.14	2.24
Glycine		0.85	0.91	0.89	0.87	0.88
Proline		0.74	0.77	0.80	0.72	0.72
Serine		0.71	0.71	0.76	0.72	0.72
Tyrosine		0.43	0.43	0.44	0.44	0.40

^1^ Origins of defatted rice bran within China. ^2^ SDF, soluble dietary fiber; IDF, insoluble dietary fiber.

**Table 2:** Composition and nutrient contents of experimental diets (as-fed basis).

Item	Origin ^1^	Heilongjiang	Heilongjiang	Jiangsu	Jilin	Liaoning	Basal Diet
	Process	Extruded	Pelleted	Extruded	Extruded	Extruded	
Ingredients, %							
Corn		49.58	49.58	49.58	49.58	49.58	71.67
Soybean meal		17.26	17.26	17.26	17.26	17.26	24.95
DFRB ^2^		30.00	30.00	30.00	30.00	30.00	-
Dicalcium phosphate		0.90	0.90	0.90	0.90	0.90	0.90
Limestone		0.90	0.90	0.90	0.90	0.90	0.90
Salt		0.35	0.35	0.35	0.35	0.35	0.35
Vitamin/mineral premix ^3^		0.50	0.50	0.50	0.50	0.50	0.50
Lysine-HCl		0.35	0.35	0.35	0.35	0.35	0.50
_DL_-Methionine		0.05	0.05	0.05	0.05	0.05	0.07
_L_-Threonine		0.11	0.11	0.11	0.11	0.11	0.16
Analyzed composition, %							
Gross energy, MJ/kg		15.59	15.59	15.81	15.71	15.80	15.85
Dry matter		86.78	86.78	87.08	86.97	87.22	85.96
Crude protein		16.18	17.22	14.80	15.48	15.97	16.20
Neutral detergent fiber		17.04	19.19	17.17	13.81	15.86	11.27
Acid detergent fiber		7.53	8.92	7.29	5.78	6.55	4.28
Ash		6.64	7.18	6.40	6.34	6.26	4.52

^1^ Origins of defatted rice bran within China. ^2^ DFRB, defatted rice bran. ^3^ Premix provided the following per kg of diet for pigs: vitamin A, 5512 international unit (IU); vitamin D_3_, 2200 IU; vitamin E, 30 IU; vitamin K_3_, 2.2 mg; vitamin B_12_, 27.6 μg; riboflavin, 4 mg; pantothenic acid, 14 mg; niacin, 30 mg; choline chloride, 400 mg; folacin, 0.7 mg; thiamin, 1.5 mg; pyridoxine, 3 mg; biotin, 44 μg; Mn (MnO), 40 mg; Fe (FeSO_4_·H_2_O), 75 mg; Zn (ZnO), 75 mg; Cu (CuSO_4_·5H_2_O), 25 mg; I (KI), 0.3 mg; Se (Na_2_SeO_3_), 0.3 mg.

**Table 3:** Effect of diet on digestibility coefficients and nitrogen balance in growing pigs.

Item ^3^	Origin ^1^	Heilongjiang	Heilongjiang	Jiangsu	Jilin	Liaoning	Basal Diet	SEM ^2^	*p*-Value
	Process	Extruded	Pelleted	Extruded	Extruded	Extruded			
Body weight, kg		35.69	34.05	35.52	36.60	34.73	35.90	1.33	
Dry matter intake, kg		1.37	1.25	1.34	1.35	1.31	1.34	0.07	0.93
Digestibility coefficients, %									
Dry matter		78.76 ^ab^	75.39 ^b^	79.35 ^ab^	81.72 ^ab^	81.06 ^ab^	86.22 ^a^	1.73	<0.01
Gross energy		80.97 ^ab^	78.19 ^b^	80.95 ^ab^	83.51 ^ab^	82.66 ^ab^	86.36 ^a^	1.53	<0.05
Crude protein		79.23 ^ab^	80.03 ^ab^	76.56 ^b^	80.52 ^ab^	80.06 ^ab^	84.82 ^a^	1.72	<0.05
Neutral detergent fiber		60.41	54.98	61.46	59.23	60.63	61.18	3.91	0.86
Acid detergent fiber		58.82	50.81	57.61	56.42	55.55	59.96	4.38	0.74
Organic matter		81.95 ^ab^	78.85 ^b^	82.09 ^ab^	84.58 ^ab^	83.79 ^ab^	87.85 ^a^	1.48	<0.05
Nitrogen balance, g/day									
Intake		41.07	37.19	35.61	39.11	39.76	40.44	1.85	0.30
Fecal output		8.79 ^a^	8.09 ^ab^	8.19 ^a^	7.22 ^ab^	7.33 ^ab^	6.13 ^b^	0.64	<0.05
Urinary output		7.41	5.75	4.26	6.49	6.15	4.60	1.05	0.30
Retention		24.87	23.35	23.16	25.40	26.28	29.71	2.06	0.28

^1^ Origins of defatted rice bran within China. ^2^ SEM, standard error of the mean. ^3 a, b^ Different superscript within a row means significant different (*p* < 0.05).

**Table 4:** Effect of diet on energy balance of growing pigs.

Item ^2^	Origin ^1^	Heilongjiang	Heilongjiang	Jiangsu	Jilin	Liaoning	Basal Diet	SEM	*p*-Value
	Process	Extruded	Pelleted	Extruded	Extruded	Extruded			
Energy balance, kJ/kg BW^0.6^/day									
ME intake		2234	2079	2217	2193	2237	2398	106.41	0.49
Total heat production		1202	1181	1212	1163	1218	1277	44.40	0.34
Adjusted total heat production		1205	1197	1216	1170	1220	1265	38.85	0.78
Fasting heat production		746	726	753	726	755	773	38.27	0.67
Retained energy (RE), kJ/kg BW^0.6^/day									
RE_P_		433	421	422	437	461	516	32.27	0.30
RE_L_		672	471	525	616	606	605	93.61	0.71
Total		1105	892	947	1053	1067	1121	111.19	0.65
Respiratory quotient (RQ)									
Fed state		1.09	1.09	1.06	1.06	1.07	1.11	0.02	0.17
Fasted state		0.81	0.80	0.83	0.82	0.80	0.81	0.02	0.94
Energy utilization, %									
ME/DE		96.17 ^a^	96.83 ^a^	96.67 ^a^	93.67 ^b^	95.17 ^ab^	96.62 ^a^	0.01	0.05
NE/ME		81.33	81.33	81.00	78.83	79.50	80.17	0.03	0.97
Energy values, MJ/kg DM									
DE		14.51 ^bc^	13.79 ^c^	14.71 ^bc^	15.11 ^ab^	15.18 ^ab^	15.89 ^a^	0.31	<0.01
ME		13.95 ^bc^	13.32 ^c^	14.23 ^bc^	14.15 ^bc^	14.47 ^ab^	15.29 ^a^	0.34	<0.01
NE		11.36	10.85	11.49	11.15	11.51	12.23	0.46	0.44

^1^ Origins of defatted rice bran within China. ^2^ Data are means of 6 observations. BW, body weight; DM, dry matter; DE, digestible energy; ME, metabolizable energy; NE, net energy; RE_P_, retained energy as protein; RE_L_, retained energy as fat; SEM, standard error of the mean. ^a-c^ Different superscript within a row means significant different (*p* < 0.05).

**Table 5:** Energy utilization and energy value of the five ingredients (DM basis).

Item ^2^	Origin ^1^	Heilongjiang	Heilongjiang	Jiangsu	Jilin	Liaoning
	Process	Extruded	Pelleted	Extruded	Extruded	Extruded
Digestibility coefficients, %						
Gross energy		68.55	59.30	68.92	78.59	74.25
Crude protein		62.41	55.63	58.35	71.90	68.91
Neutral detergent fiber		74.60	76.79	69.75	75.58	75.46
Acid detergent fiber		59.91	51.94	61.65	58.68	60.18
Organic matter		57.12	36.86	54.11	53.39	48.98
Energy utilization, %						
ME/DE		0.96	0.98	0.98	0.95	0.93
NE/ME		0.77	0.82	0.83	0.78	0.79
Energy values, MJ/kg DM						
DE		11.17	8.16	12.50	13.83	13.90
ME		10.76	8.02	12.25	13.14	12.95
NE		8.24	6.56	10.15	10.22	10.17

^1^ Origins of defatted rice bran within China. ^2^ DM, dry matter; DE, digestible energy; ME, metabolizable energy; NE, net energy. Data are means of 6 observations.

## Data Availability

Data is contained within the article. The data presented in this study are available in Evaluation on Net Energy of Defatted Rice Bran from Different Origins and Processing Technologies Fed to Growing Pigs.

## Abbreviations

AA, amino acid; ADF acid detergent fiber; ATTD, apparent total tract digestibility; BW, body weight; CP, crude protein; DE, digestible energy; DFRB, defatted rice bran; DM, dry matter; EE, ether extract; FHP, fasting heat production; GE, gross energy; HP, heat production; IDF, insoluble dietary fiber; IU, international unit; ME, metabolizable energy; NDF, neutral detergent fiber; NE, net energy; OM, organic matter; RE, Retained energy; RE_P_, Retention of energy as protein; RE_L_, Retention of energy as lipid; RQ, respiratory quotient; SDF, soluble dietary fiber; SID, standardized ileal digestible; TDF, total dietary fiber.
